# Supplementary material for: Magnitude of neonatal meningitis and associated factors among newborns with neonatal sepsis admitted to the University of Gondar Comprehensive Specialized Hospital, North Gondar, Ethiopia
Source: PLoS One. 2023 Sep 12;18(9):e0290639. doi: 10.1371/journal.pone.0290639 (PMC10497136; doi:10.1371/journal.pone.0290639)
Supplement: Attachment — Submitted filename: Response to reviewers-meningitis.docx [file pone.0290639.s002.docx]

**A point-by-point response to editors’ and reviewers’ comments**

**To the Editorial team**

1. Please ensure that your manuscript meets PLOS ONE's style requirements, including those for file naming.

Answer:- Thank you for the comments. We have corrected the manuscript based on PLOS ONE's style requirements

2. Your ethics statement should only appear in the Methods section of your manuscript.

Answer: - Noted, thanks. We have moved the ethical statement to the methods section

3. Please include captions for your Supporting Information files at the end of your manuscript, and update any in-text citations to match accordingly

Answer:-Noted, we have included all the information in the manuscript.

**Reviewer 1**

Manuscript Title: Higher prevalence of neonatal meningitis in early-onset sepsis than late-onset among newborns admitted to the University of Gondar Comprehensive Specialized Hospital, Ethiopia

General

1. Is the manuscript technically sound, and do the data support the conclusions?

Yes, the manuscript is technically sound that has used data to support the conclusion

Response: Thank you

2. Has the statistical analysis been performed appropriately and rigorously?

Yes, but the regression models can be reduced by deleting non-significant data see table 4

Response:- Thank you for the suggestion. We have corrected table 4

3. Have the authors made all data underlying the findings in their manuscript fully available?

Yes, it is available

Response:- Thank you

4. Is the manuscript presented in an intelligible fashion and written in Standard English?

Yes, it is written in Standard English but needs corrections and editions throughout the process

Response; - The language has been edited.

5. Review Comments to the Author
I. Title modification–

Answer:- We have acknowledged the suggestion and the title has been modified.

II. Abstract -avoid abbreviations from the abstract see line 36-37 of the abstract e.g., EONS, LONS, and ROM

Answer:-We appreciated the comments and abbreviations are avoided from the abstract

III. Keywords- add a few words like neonatal meningitis

Answer:-we have added the suggested words in the keywords.

IV. Introduction- Either write fully for the first time or do not use too much abbreviations’ in the txt see line 60 GBS and 66 CSF etc.

Answer:- we have corrected it

V. Methods

- The single population proportion formula is ok but the sample size failed to consider the correction formula

Answer:-We used single proportion formula and a correction formula was applied.

Considering the P-value as 17.9% from a previous study in Kenya. So the sample size was calculated using the following formula;

n= (za/2 )2*p(1-p)/d2

n=(1.96)2*0.179(1-0.179)/0.05*2

n=225, but since the source population is <10,000 the final sample size calculated as

nf =ni/(1+ni/N)

nf =225/(1+225/800)

nf =176

Adding 5% for non-respondents, **the final size will be 185**

n=desired sample

ni= initial sample

nf=final sample

- Was it convenient to select every third from the total population of 540?

Answer:-We understood why the question was raised but the data collectors were well trained and the investigators supervise the situations. The selection was not that much a problem.

- What was the problem when you excluded the incomplete documentation

Response:- It might reduce the statistical power of the study and the study outcome if the missing data is more than 10% of the total.

- Data collection and procedure;- It would be nice if you indicate where the pretest was conducted.

Answers:- Pretest was done in 5% of the calculated sample size in another area at Debre tabor Hospital, Ethiopia. Then, the necessary modifications were done based on the analysis. It’s revised in the manuscript as well.

VI. Results

- The calculated value was 185 and the response rate became 171(92%) of the total population. The contingency added in the calculated sample size was 5%; however, the response rate is 92% which is more than the contingency added. What is the justification for this problem?

Answer: We have accepted the comments and noticed the problem. It has been good if we used at least 10% contingency as a backup. Generally, if the response rate is >90%, the outcome of the study is considered adequate.

- Table 1: Socio-demographic factors: Family income in this result is only 103, what is the justification and what is the income of 68 individuals?

Response:- thank you. It was a type error and corrected

- Table 2: Maternal and neonatal health-related characteristics

ROM total population is 162 justifications for this and the loss of 9 participants

Response:- thank you. It was a type error and corrected

- Table 3: Clinical Manifestations of the study participants

This table is not clear either redefine or make it clear

Response:- it is important to mention, how many meningitis cases have these individual symptoms.

- Figure 1 needed to add percentages on the top of each bar

Answer:- we have corrected it

- Table 4: Factors associated with neonatal meningitis: GA Row 1 is not significant and Birth weight is not significant are not significant is so we do not have to overcrowd the table
- Answer:- We appreciate the comments and corrected them accordingly.

VII. Discussion:

- Minimize the discussion and focus on the main points

Response:-Thank you. We have tried to minimize the discussion and focused on the main finding of the study.

- Line 157: Ethical considerations

What do you mean by “Written informed consent was obtained from the parents of the study participants after the purpose of the study was explained?”

Response:- For those who were able to read and write –they signed and continue the interview. For those who were unable to read and write, the interviewer read the consent form and if they agreed, they signed with their fingerprint and continue the interview.

**Reviewer 2**

1. I suggest the authors modify the title to, “Prevalence of neonatal meningitis and associated factors among newborns admitted to the University of Gondar Comprehensive Specialized Hospital, Ethiopia”

Response: We appreciated the detailed comments and we have modified the title according to the suggestion

2. The target population must be consistent throughout the document. Eg. In the title, your target populations are “newborns admitted to the University of Gondar Comprehensive Specialized Hospital”.

Response:- Thank you for the plausible observation. We have corrected it accordingly.

3. **Abstract**

- Remove all the abbreviations

Response:- We have removed all the abbreviations.

- Methods

In the method section, key information such as sample size, sampling technique, and software used for data entry are missed

Answer:- Thank you for the comments, we have included.

- When you report the prevalence of neonatal meningitis, you include the 95% confidence interval.

Answer:- We have included the CI

- The conclusion is almost a copy of the result section and it lacks recommendations.

Answer:- We appreciated the comments and corrected them accordingly.

4. **Introduction**
i. The introduction does not tell us why this study was conducted. You tried to mention some information starting from lines 70 & 71 as “to our knowledge, there is no data that studied the difference in magnitude to rationalize this clinical practice in most developing countries like Ethiopia” but as you cited in Ethiopia there is “a 10-year retrospective review on Neonatal meningitis”. What new information do we get from this study?

Answer:- Thank you for the comments. The current study is different from the previous study in Ethiopia (The 10-year retrospective study on Neonatal meningitis at tikur ambessa hospital, Ethiopia). Neonatal meningitis was defined in the previous study based on only CSF culture. The study population was not restricted to newborns with neonatal sepsis, which also included other diagnoses that need CSF analysis like (breast abscess, cellulitis, and severe pneumonia….etc

The current study used broader and more practicable criteria to define neonatal meningitis (cell count, gram stain, culture, glucose, protein…), studied associated factors, clinical presentations, and rationalized the indication for neonatal meningitis.

ii. The introduction fails to address previously proposed national or local solutions to minimize the morbidity and mortality associated with the problem under study (neonatal meningitis).

Answers; - Thank you for the comments. As we have mentioned in the introduction, the indications for obtaining cerebrospinal fluid in the evaluation of meningitis in sepsis are controversial in most setups including in our country. In our setup, we used textbook recommendations and expert opinions for the indication of CSF analysis. We will do lumbar puncture for all neonates with LONS (not rationalized), positive neurologic symptoms (lethargy, seizure- low sensitivity of symptoms), positive blood culture (low sensitivity), and maternal chorioamnionitis in EONS. We believed that some of the indications are not rationalized especially the postnatal age. So our research answered appropriately for the local and national recommendations.

5. **Methods**

- There are some contradictory ideas in the method section. In line 76, you write as the study is prospective on the other hand in the exclusion criteria (line 92) you exclude cards with incomplete documentation. One advantage of the prospective study is to overcome such problems so why do you exclude them? In addition, you exclude “neonates whose families or legal guardians refused to participate”, which is not the right approach rather you have to consider those as non-responders.

Response:- Thank you for the observation. We approached the participants at the time of the interview and review the charts at the same time. If we missed important information in the chart, then we considered it as incomplete and excluded from the study. We have accepted the comments for the non-responders and corrected them accordingly

- **Sample size**. First, why did you take the proportion from Kenya despite there being a study in Ethiopia?
- Response:-Thank you for the important comments

We didn’t take the proportion from the study in Ethiopia because neonatal meningitis was not practically defined (they only used culture-confirmed meningitis-very narrow definition) and the study population is not the same as our study population. We took the Kenyan study because our study has a similar definition, objective, and study population with the Kenyan study. We also related geographically.

- Second, in such rare proportions, as a right thumb rule, we have to decrease the margin of error or determine the sample size using the factors to get an appropriate sample size. In this study, the sample size is 185, which is not enough for logistic regression. Even when you look at the regression table, the confidence intervals are too wide, which may be due to the small sample size.

Response;_ We understood why the question raised

Actually, we used the single proportion formula, and further correction formula was applied to reach the final sample size of 185. Probably be good if we did not use the correct formula, and add 10% contingency, we would have near to 250 sample size which will have powerful research. Still, a sample size of 185 is okay, for rare diseases to have a conclusion. The associated variables also passed the 2by2 table in chi-squire. So the outcome of the study is reliable and justifiable.

The data collection method is poorly written. First, there is no clear information about the data collection tool: validity, reliability, means of diagnosis… Second, how many data collectors were involved? Their experience. Which data collection method was used, interviewer-administered, chart review, or both?

Response: Thank you for the comments. We have corrected it accordingly in the manuscript. The data collectors are pediatric residents and are able to provide neonatal care in the NICU.

We used both interviewer-administered and chart review for data collection tools.

- Replace multivariate with multivariable; there is a big difference between them.

Response:- thank you. We have corrected it.

- I have doubts about your operational definition. If the newborn is admitted to NICU with suspected sepsis empirical management using antibiotics may be initiated, and the CSF analysis is sent if they are unresponsive to the medication. CSF analysis after the initiation of antibiotics is not accurate and has a significant effect on the overall prevalence of meningitis. What is your justification for this?

Response:- We understood why you raised the question but as much as possible we will take CSF analysis before the initiation of antibiotics. If the patients took antibiotics before the LP, then we will use other criteria like cell count, protein, and latex agglutination to diagnose meningitis

6. **Results**

- In line 143, most (99%) newborns had normal Apgar 144 scores>7. First, which minute Apgar score is it? Second, do you think newborns with suspected sepsis are presented with such an Apgar score?

Response; We have used the 5^th^ minute APGAR score if they delivered in our hospital and APGAR indirectly assessed whether they cried or not if they were delivered in other hospitals.

APGAR in sepsis might be affected if the infection occurred before delivery. Otherwise, if the infection occurred at the time of delivery or after delivery, we don’t expect the APGAR score to be affected.

- It is unclear how did you assess some variables such as maternal chorioamnionitis, Maternal UTI, ROM, and even GA. To get reliable information about them the mother must deliver them to the same hospital where the study was conducted. However, there may be referral cases, so how could you get such information? Was it self-report?
- Response:- We have used both data collection methods–chart review, diagnosis at delivery, or self-report from the mother during pregnancy.
- All the information written under the result section must be based on the data collection tool and procedure mentioned under the method section. You report the blood culture and sensitivity results despite nothing being said in the method section.

Response:- Thank you for the comments. We include blood culture as one associated factor which might be related to neonatal meningitis. In previous studies, positive blood culture is associated with neonatal meningitis (we have discussed it in the introduction).

- The detail of the CSF analysis is also missed in the method despite your report in the result section (i.e. “28(85%) was diagnosed with cell count, 4(12.1%) with gram stain, and one case by CSF culture”)

Response:- Thank you for the suggestion. We have included in the operational definitions of meningitis based on cell count, gram stain, glucose…etc.

- The 95% CI is mandatory for your discussion. In addition, you write as “meningitis was higher in EONS 22.8% than in LONS, which was 16.8%

Response:- Thank you for the observation. We accepted the comments and corrected them accordingly.

- The final model must be computed after including sepsis (early vs late) as one factor.
- Response:-We have computed SPSS (as EONS VS LONS) as a factor for neonatal meningitis. The result showed no statistically significant association.
- The discussion, conclusion, and recommendation must be modified after updating the model.

Response: As we have discussed, the model was not updated because the postnatal age (EONS VS LONS) was not a statistically significant factor for neonatal meningitis.
