# Supplementary material for: Magnitude of neonatal meningitis and associated factors among newborns with neonatal sepsis admitted to the University of Gondar Comprehensive Specialized Hospital, North Gondar, Ethiopia
Source: PLoS One. 2023 Sep 12;18(9):e0290639. doi: 10.1371/journal.pone.0290639 (PMC10497136; doi:10.1371/journal.pone.0290639)
Supplement: Attachment — Submitted filename: A point by point response - new meningitis.docx [file pone.0290639.s003.docx]

**A point-by-point response to editors’ and reviewers’ comments**

**To the Editorial team**

**Answer- We thanks the editorial team for giving us the opportunity to revise our manuscript.**

**REVIEWER 1**

1. **If the authors have adequately addressed your comments raised in a previous round of review and you feel that this manuscript is now acceptable for publication, you may indicate that here to bypass the “Comments to the Author” section, enter your conflict of interest statement in the “Confidential to Editor” section, and submit your "Accept" recommendation.**

**Reviewer #1: All comments have been addressed**

**Answer- Thank you.**

2. **Is the manuscript technically sound, and do the data support the conclusions?**

**Reviewer #1: Yes**

**Answer- Noted, Thanks.**

3. **Has the statistical analysis been performed appropriately and rigorously?**

**Reviewer #1: Yes**

**Answer- We appreciated it, thank you.**

4. **Have the authors made all data underlying the findings in their manuscript fully available?**

**Reviewer #1: Yes**

**Answer- Thank you**

5. **Is the manuscript presented in an intelligible fashion and written in Standard English?**

**Reviewer #1: Yes**

**Answer- Thank you, Noted.**

6. **Review Comments to the Author**

**Reviewer #1: All comments are incorporated and corrected from my side and I have seen what the authors responded to my previous concern and comments. Thus, I accept to proceed to the next step**

**Answer- We really appreciated your comments, thank you again.**

**REVIEWER 1**

1. **If the authors have adequately addressed your comments raised in a previous round of review and you feel that this manuscript is now acceptable for publication, you may indicate that here to bypass the “Comments to the Author” section, enter your conflict of interest statement in the “Confidential to Editor” section, and submit your "Accept" recommendation.**

**Reviewer #2: All comments have been addressed**

**Answer- Appreciated, Thank you**

2. **Is the manuscript technically sound, and do the data support the conclusions?**

**Reviewer #2: Yes**

**Answer- Noted, Thank you.**

3. **Has the statistical analysis been performed appropriately and rigorously?**

**Reviewer #2: No**

**Answer- Thank you for your genuine response, we have gone through the details of of the statistical analysis, and critical model evaluation was done before we evaluate the final output of the binary logistic regression. Here below presented, the details of the model evaluation( see last page).**

4. **Have the authors made all data underlying the findings in their manuscript fully available?**

**Reviewer #2: Yes**

**Answer- Thank you**

5. **Is the manuscript presented in an intelligible fashion and written in Standard English?**

**Reviewer #2: No**

**Answer: -** **The language has been revised and edited.**

6. **Review Comments to the Author**

**Reviewer #2:**

**In …., why do you only display significantly associated variables in table 4? Display all the variables included in the model with their respective measure of effects both in the bivariable and multivariable column.**

**Answer-Thank you for the observation, we removed the non-significant variables from the table 4 is based on the comment from reviewer 1. We also believed that all variables having p-value < 0.2 in bivariable logistic regression should be presented in multivariable regression table as well. We have included those variables in table 4.**

**The majority of the concerns raised in the previous review were addressed but still the justification given for the final model needs further clarification.**

**Answer- Here is the details about the statistical analysis and model evaluation;**

**We first evaluate all the variables by bivariable logistic regression, and then the p-value was checked. Only four variables were having p-value less than 0.2 which are gestational age, birth weight, duration of labor, and rupture of membrane. Then all the four variables were computed in multivariable logistic regression. Then before we evaluate the output equation, we have checked the tests and model evaluation by using the following tests:-**

1. **The omnibus test of model coefficients**

**In this model if the p-value is less than 0.05, then the researchers have significant model that should be interpreted further. In our case, the p-value was 0.01, so it passed this model.**

2. **Hosmer and Lemeshow test**

**In this test, if the p-value is >0.05, the model does fit the data and should be further interpreted. In our paper, the p-values was 0.76, so it passed this test.**

3. **Overall accuracy of the model**

**In this model, the total accuracy of the model should be at least 80%. In our paper, the overall accuracy of the model was 84.8%, which also passed this model.**

**Finally we evaluate the outputs of multivariable logistic regression in the variables and interpreted accordingly with the values.**
